# Unveiling natural anti-inflammatory compounds for sinusitis treatments by modulating FOXP3, C5aR1, and LIF

**DOI:** 10.3389/fimmu.2026.1792658

**Published:** 2026-06-17

**Authors:** Chengjian Cao, Md Arefin Hossen, Md Sahriyer Efti, Ahmed A. Warda, Soad K. AlJaouni, Ali ElFar, Chaoxiang Lv

**Affiliations:** 1 Zigong Academy of Medical Sciences, Zigong First People’s Hospital, Zigong, China; 2 Head and Neck Surgery, Otolaryngology. Southwest Medical University Affiliated Hospital, Luzhou, China; 3 Oncology, The Research Center for Medicine, Southwest Medical University, Luzhou, China; 4 Chemistry Department, Faculty of Science, Suez Canal University, Ismailia, Egypt; 5 Department of Hematology/Pediatric Oncology, Yousef Abdulatif Jameel Scientific Chair of Prophetic Medicine Application, Faculty of Medicine, King Abdulaziz University, Jeddah, Saudi Arabia; 6 Key Laboratory of Epigenetics and Oncology, The Research Center for Preclinical Medicine, Southwest Medical University, Luzhou, China

**Keywords:** C5aR1, chronic rhinosinusitis, Foxp3, LIF, natural compounds

## Abstract

Chronic rhinosinusitis (CRS) is a condition with a significant healthcare and economic burden, with a prevalence of 12% in the West. The existing therapies mainly attenuate downstream inflammatory mediators but do not address the underlying immune-regulatory abnormalities that drive disease recurrence and lifelong treatment. Evidence has shown that a regulatory axis involving forkhead box P3 (FOXP3) + regulatory T cells (Tregs), the complement component 5a receptor 1 (C5aR1), and leukemia inhibitory factor (LIF) is crucial for regulating immune tolerance in sinonasal tissues. This review discusses the mechanistic basis for modulating FOXP3, C5aR1, and LIF with natural anti-inflammatory products for the treatment of CRS. A comprehensive literature review across PubMed, Scopus, and Web of Science (2000–2026) was conducted to identify research articles on the molecular mechanisms, preclinical evidence, and clinical application of natural compounds in inflammatory disorders and the sinonasal inflammatory pathway. Various studies show that FOXP3+ Tregs are highly depleted in CRS with nasal polyps. Besides, complement signaling via C5aR1 prevents active Treg induction by activating the PI3K-AKT-mTOR pathway. LIF enhances the expression of FOXP3 and opposes Th17 polarization caused by IL-6. Natural products such as epigallocatechin-3-gallate, curcumin, and rosmarinic acid regulate pathways by inhibiting DNA methyltransferases, the complement cascade, and JAK-STAT signaling. Bromelain and cineole have clinical evidence in favor of efficacy in acute sinusitis, and other compounds are showing emerging evidence. The FOXP3-C5aR1-LIF axis is a mechanistic therapeutic target that is largely overlooked by the current biologics. Natural compounds offer potential benefits, including multi-target activity, favorable safety profiles, and the ability to modulate the upstream immune system. Phenotype-based CRS populations should be studied in well-designed trials, bioavailability formulations should be optimized, and synergistic combinations should be systematically explored.

## 1 Introduction

Chronic rhinosinusitis (CRS) is one of the most common chronic inflammatory diseases in the industrial world, with a 12-16% prevalence and direct healthcare costs of over $8 billion every year in the United States (1) and affecting an estimated 5–12% of the general population across diverse geographic regions, with particularly high prevalence documented in Europe, North America, and parts of Asia (2, 3). The illness affects the quality of life in the same way as chronic obstructive pulmonary disease and congestive heart failure (4). The clinical definition of CRS is inflammation of the sinonasal tissue lasting more than 12 weeks, accompanied by cardinal symptoms such as nasal congestion, nasal discharge, facial pain, and loss of smell (5, 6).

Modern knowledge accepts CRS as being heterogeneous, stratified on inflammatory endotypes (7). In the Western population, CRS with nasal polyps (CRSwNP) is type 2 inflammation, which is eosinophilic and is dominated by Th2 cytokines (interleukin (IL)-4, IL-5, and IL-13), while Type 1 (Th1/IFN-g) and Type 3 (Th17/IL-17) are all non-Type 2 inflammation (8–12). Existing therapeutic options, such as intranasal corticosteroids, systemic corticosteroids in the case of an exacerbation, and Type 2 inflammatory (dupilumab, omalizumab, and mepolizumab) biologic agents, have transformed the management of severe CRSwNP but have significant limitations: they treat downstream effects but do not restore the basis of immune tolerance, which leads to the disease recurring when the therapy is not maintained (13–16). For some patients with CRSwNP, biologics are a new treatment option recommended in the guidelines. Some of the caveats include the real-world access and the option of surgery, and there is a lack of direct studies comparing the drugs (17). In the future, therapy will be tailored to the individual, using a combination of biologics and surgery as needed.

CRS is defined by an underlying cause of immune tolerance, involving dysfunction of regulatory T cells (Tregs) (18). Forkhead box P3 (FOXP3) + Tregs are key regulators of immune homeostasis in a variety of ways: cell-contact-mediated suppression via cytotoxic T-lymphocyte-associated protein-4, lymphocyte-activation gene-3, IL-10, and transforming growth factor-beta (TGF-*β*) secretion, and metabolic competition by consuming IL-2 and stimulating the development of tolerogenic dendritic cells (19). Various reports indicate that Tregs are significantly deficient in nasal polyps: FOXP3+ cells were found to be lower in controls (6.16 ± 2.75 vs 11.33 ± 9.9 cells/high power field (HPF), *p* < 0.05) (20). More importantly, Tregs’ migration to the airway epithelium is hindered, indicating both numerical and functional defects.

The complement system is considered a determinant in the pathogenesis of CRS (21). Increased levels of complement activation products, such as the C5b-9 membrane attack complex, C4d, and activated C1, have been shown to occur in nasal polyp tissue (22). Furthermore, C1 activation levels showed the strongest correlation with local immunoglobulin and C5a levels. These findings collectively indicate that the classical pathway is a key contributor to complement activation in patients with CRS (23). Interestingly, one of the most critical studies demonstrated that C3aR and C5aR signaling in CD4+ T cells directly inhibits Treg induction. Complement receptor stimulation activates the phosphatidylinositol 3-kinase (PI3K)-protein kinase B (AKT)-mechanistic target of rapamycin (mTOR) pathway (24).

Leukemia inhibitory factor (LIF) is an IL-6 family cytokine, which has a rather protective and anti-inflammatory effect in respiratory tissues (25). Most appropriately to CRS, LIF enhances FOXP3 expression and the Th17-inhibitory effects of IL-6 (26). TGF-*β* and IL-6 induce extensive FOXP3 repression and initiate RAR-related orphan receptor-gamma (RORγ)t-mediated Th17 gene expression (27, 28). The IL−6−induced Th17 cultures and consequent IL−17A release are significantly dampened by the addition of LIF (26, 29), suggesting that modulating the LIF/IL−6 axis could be used as a therapeutic approach to correct the prevalent inflammation. The mechanistic interrelation provides compelling reasons for therapeutic intervention: the high expression of C5aR1 drives complement signaling, which inhibits Treg induction; the absence of FOXP3 allows uncontrolled Th2/Th17 responses; the imbalance between IL-6 and LIF further suppresses regulatory function (24, 30, 31).

**Figure 1:**
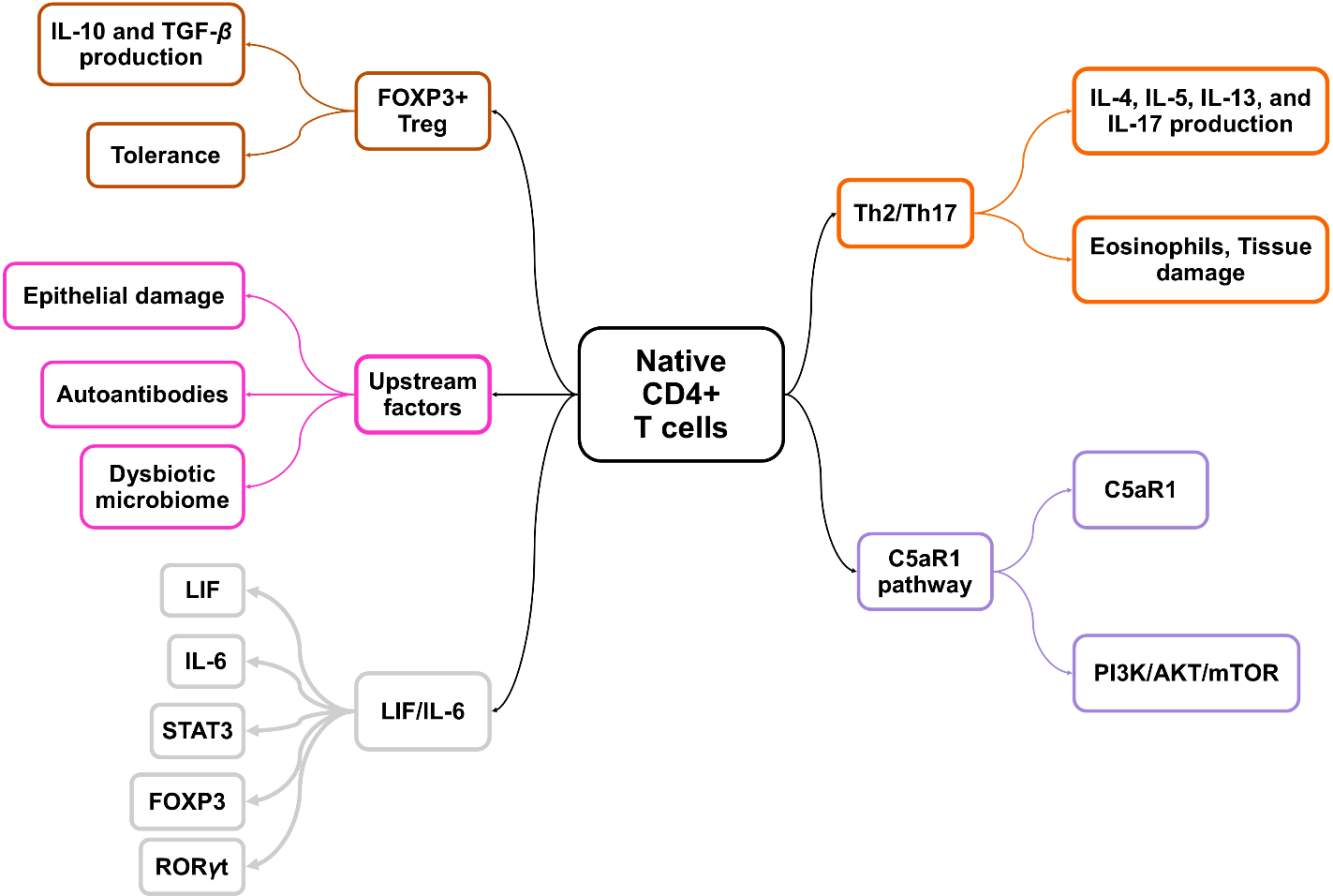
Systems biology network showing forkhead box P3 (FOXP3)-complement component 5a receptor-1 (C5aR1)-leukemia inhibitory factor (LIF) interactions. IL, interleukin; ROR*γ*, RAR-related orphan receptor-gamma; STAT3, signal transducers and activators of transcription 3; TGF-*β*, transforming growth factor-beta.

The interaction between FOXP3, C5aR1, and LIF is a key pathogen of chronic inflammation in CRS (**Figure 1**). Recent discoveries have shown that C5aR1 signaling directly suppresses FOXP3+ regulatory T cell activity, favoring a pro-inflammatory effect. At the same time, the new positive feedback mechanism, with this C5aR1-driven inhibition enhanced by increased local LIF concentration, further imbalances the immune response. This combined axis provides a novel mechanistic understanding of the immune dysfunction of CRS, especially treatment-resistant phenotypes (8, 11, 32).

Theoretically, natural anti-inflammatory compounds have many benefits: they act on multiple targets or affect multiple pathways rather than a single molecule; they are often inexpensive; they are generally safe; they are immune-modulatory; and they are less immunosuppressive than biologic compounds. This review critically analyzes the mechanistic rationale and evidence supporting the involvement of FOXP3, C5aR1, and LIF as possible targets for natural compounds in sinusitis therapies.

## 2 Molecular mechanisms and network integration

### 2.1 FOXP3 and regulatory T cell deficIency

FOXP3 is a transcription factor that acts as the master regulator of Treg development and function (33). The non−redundant requirement for FOXP3 was clearly established when loss−of-function mutations were shown to cause the severe autoimmune multisystem condition IPEX syndrome (immune dysregulation, polyendocrinopathy, enteropathy, X−linked), which necessitates hematopoietic stem cell transplantation (34). There is substantial evidence that FOXP3 is a transcriptional activator and repressor in physiological settings, binds to more than 700 gene loci, and indirectly influences thousands more through chromatin remodeling, yet very little evidence has been gathered in humans or mice sinonasal tissue.

So far, there is no direct evidence that FOXP3-induced chromatin remodeling occurs in polyps in the context of CRS. There are several indirect observations suggesting that the transcriptional regulatory machinery is disrupted; however, quantitative deficiency of FOXP3^+^ cells in polyps is well documented (20). The altered migration of polyp-derived Tregs to the airway epithelium also points to functional abnormalities in addition to their reduced numbers. Thus, the mechanisms underlying Treg deficiency in CRS are likely to include reduced local IL−2 production, imbalanced signaling through the TGF−*β* receptor, disruption of the epithelial barrier, and, most significantly, activation of the complement pathway through C5aR1, in the context of which FOXP3 expression is epigenetically limited.

### 2.2 C5aR1 Direct suppression of Treg induction

C5a-C5aR1 triggers PI3K, which in turn phosphorylates AKT and triggers mTOR (24). The FOXO transcription factors, FOXO1 and FOXO3a, are directly phosphorylated by activated AKT, which then targets them for cytoplasmic degradation by the ubiquitin-proteasome system. FOXO1 and FOXO3a have been shown to play vital roles in binding to the FOXP3 promoter and driving FOXP3 transcription; their inactivation is an important molecular pathway through which C5aR1 signaling attenuates FOXP3 induction (35–37). Besides, active mTOR suppresses TGF-*β*-induced FOXP3 transcription by phosphorylating transcription factors needed to activate FOXP3 promoter - a cell-intrinsic inhibition of Treg differentiation (38, 39). In the absence or blockage of C5aR1 signaling, naive CD4+ T cells transform into FOXP3+ induced Tregs by an auto-inductive mechanism: early TGF-*β* stimulation causes FOXP3, these early Tregs make further TGF-*β*, creating feed-forward loops, and induced Tregs are more stable. Exposure to C5a disrupts this cycle and facilitates Th17 differentiation (24). This mechanism produces a microenvironment actively antagonistic to Treg induction in CRSwNP, characterized by high C5aR1 expression and complement activation. Recruited naive T cells encounter C5a, and complement receptor signals override tolerogenic cues, leading to differentiation into effector rather than regulatory phenotypes and progressive depletion of Tregs compared with effectors.

### 2.3 LIF-FOXP3 axis

While the link between the LIF-FOXP3 axis has not yet been directly demonstrated in patients with CRS or in nasal polyp tissue, compelling evidence from multiple inflammatory models supports future investigations of this axis in CRS. LIF promotes FOXP3 expression and prevents IL−6-induced Th17 development. LIF boosts FOXP3 expression by binding to the LIF receptor–glycoprotein-130 (gp130) heterodimer and activating signal transducer and activator of transcription-3 (STAT3) (26). Fortunately, the same STAT3 molecule can also be recruited by LIF to enhance FOXP3 transcription (40).

A recent proteomic study of nasal polyp tissue identified that LIF and the LIF receptor (LIFR) are both significantly upregulated in this axis in CRS. A higher LIFR level was also associated with an increased risk of postoperative recurrence (41). This does not establish a causal link between LIF and FOXP3 in CRS, but it does suggest that LIF signaling is an active pathway in sinonasal tissue and is associated with disease severity. Future studies to assess LIF and IL-6 levels in CRS tissue and functional studies using CRS-derived T cells may be required to determine whether the LIF/IL-6 balance directly affects Treg stability within the sinonasal mucosa.

### 2.4 Network-level dysregulation and therapeutic implications

CRS is observed at the network level to have a pathologic shift at all three nodes: (1) a high C5aR1/complement actively inhibits the induction of Tregs, (2) a deficiency of FOXP3+ Tregs eliminates the critical regulation of Th2/Th17 responses, and (3) an imbalance between IL-6 and LIF further suppresses regulatory functions. This model is based on well-established mechanisms observed in other inflammatory contexts; however, its direct relevance to CRS remains to be experimentally validated. This generates a positive feedback loop that sustains chronic inflammation: epithelial barrier malfunction permits allergen/microbial infiltration, which activates complement and drives Type 2 inflammation; Type 2 cytokines inhibit epithelial repair and mucociliary clearance, which are maintained by Treg deficiency; Tregs allow uncontrolled effector responses (18).

The mechanistic integration indicates that single-node targeting could turn out to be inadequate - inhibiting only C5aR1 but not IL-6 can suppress Treg recovery. But the combination C5aR1 inhibition across nodes may be synergistic: active suppression will be eliminated by C5aR1 blockage, LIF will stimulate positive signals, and direct enhancement of FOXP3 will reinforce the regulatory phenotype (24). This multi-target strategy is like effective combination therapy in other, more complicated diseases, where the dysregulation at the network level needs to be treated at the network level.

## 3 Natural compounds targeting the regulatory axis

**Figure 2:**
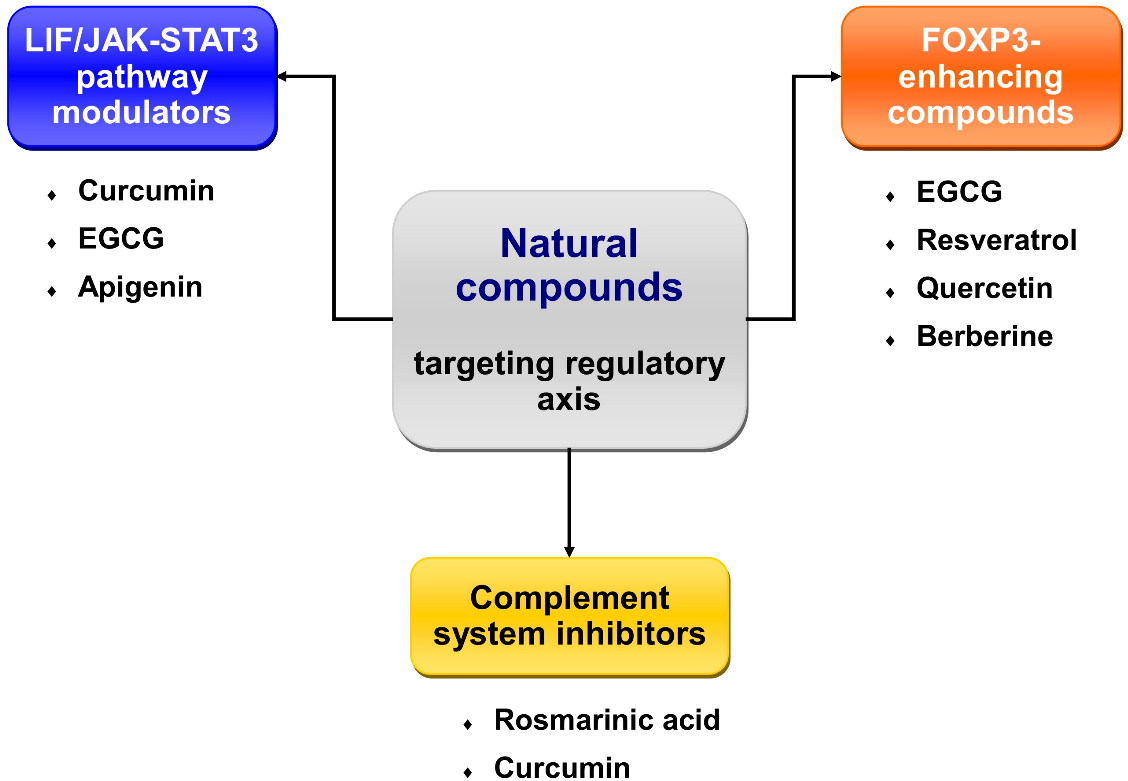
Natural compounds target the regulatory axis. EGCG, epigallocatechin-3-gallate; JAK, Janus kinase. LIF; leukemia inhibitory factor; STAT3, signal transducers and activators of transcription 3.

The direct targeting of the FOXP3-LIF-C5aR1 axis has varying evidence with the natural compounds mentioned below. Specific molecular targets, such as C3b/C5 convertase, DNA methyltransferase (DNMT), or JAK2, are mechanistically linked to the axis. In other compounds, only broad anti-inflammatory properties are described, and in some, the axis-modulating properties are assumed at the pathway level and need to be confirmed. The targeting of natural compounds to the regulatory axis is represented in **Table 1**, **Figure 2**.

**Table 1:** Key natural compounds and mechanisms.

Compounds	Primary targets	Mechanisms	Key evidence	Typical dosing	References
Berberine	FOXP3	Balancing between the pro-inflammatory Th17 and the anti-inflammatory Treg cells	Attenuating the NF-κB/JNK/RANKL signaling pathway in septic arthritis	50, 100, or 200 mg/kg	(51)
Curcumin	Improves nasal airflow and modulates the immune response	Alleviates nasal symptoms (sneezing and rhinorrhea) and nasal congestion through reduction of nasal airflow resistance	Suppression of IL-4, IL-8, and tumor necrosis factor α and increased production of IL-10 and soluble intercellular adhesion molecule	500 mg/day	(55)
Epigallocatechin-3-gallate (EGCG)	FOXP3	DNMT inhibition; IL-6 suppression	4.6-fold FOXP3 increase at 50 μM	50 mg/kg EGCG daily for 7 days	(43)
Resveratrol	FOXP3	miR-34a downregulation; AhR inhibition	Treg increase in the asthma model	100 mg/day	(47)
Rosmarinic acid	C5aR1	C3b covalent attachment; convertase inhibition	IC50 34-180 μM complement inhibition	–	(53)

In this section, *in vitro* concentrations are reported in micro molar (µM), the common unit used in cell-free and *in vitro* assays. Animal doses are reported as mg/kg of body weight, and clinical doses in humans are reported as mg/day. Since there is no direct conversion between these units without considering factors, such as oral bioavailability, protein binding, metabolic stability, etc., and allometric scaling of parameters, the values are presented in their units of measurement for clarity, and numerical comparisons should be avoided.

### 3.1 FOXP3-enhancing compounds

Epigallocatechin-3-gallate (EGCG): EGCG is a bioactive polyphenol found especially in green tea with antioxidant and anti-inflammatory properties (42). Wong et al. (43) reported that EGCG acts as a DNMT inhibitor, namely, DNMT1, DNMT3a, and DNMT3b. Transcription is blocked by methylation of FOXP3 promoter CpG islands; epigenetic silencing is removed by demethylation by EGCG. When administered at 2-50 μM (which can be reached through the consumption of green tea extracts), EGCG induced dose-dependent FOXP3 and IL-10 expression, with a 50 μM dose increasing the gene by 4.6-fold at 72 hours. *In vivo*, oral administration of 50 mg/kg/day EGCG resulted in a 1.4-fold increase in splenic Treg frequency with plasma EGCG of 7-8 μM. In conclusion, EGCG shows promise as an immune regulator by inhibiting DNA methyltransferases. It induces demethylation of the FOXP3 promoter, which enhances expression of both IL-10 and FOXP3. In both *in vitro* and *in vivo* models, EGCG may enhance Treg frequency, making green tea extract with EGCG useful for maintaining immune health and controlling inflammation.

Furthermore, EGCG reliably increases FOXP3^+^ Treg cells, inhibits Th17 responses, and restores the balance between Treg and Th17 cells across various models of systemic inflammation and asthma (44, 45). Since CRS is characterized by ongoing mucosal inflammation, impaired Treg function, and a bias toward Th17 responses, these consistent findings suggest that targeting FOXP3 with EGCG could be a highly promising approach for future research.

Resveratrol: Resveratrol, present in red grapes, has anti-inflammatory, antioxidant, anti-atherogenic, and anti-angiogenic properties (42, 46). Resveratrol improves FOXP3 in various ways, including miR-34a/miR-31 (which directly downregulate FOXP3 translation) and aryl hydrocarbon receptor (AhR)/Notch (which suppresses FOXP3 and stimulates Th17) signaling (47). Oral resveratrol enhanced CD4+ Foxp3 Tregs through FOXP3 upregulation by miR-34a in ovalbumin-induced asthma. Resveratrol has very low oral bioavailability (< 1%) because it is rapidly glucuronidated/sulfated; however, nanoemulsion preparations have much higher bioavailability and demonstrate greater activation of FOXP3. There is currently no direct evidence that resveratrol interacts with FOXP3−regulatory DNA methyltransferases or directly affects the C5aR1/LIF pathways. Instead, its influence on Treg populations probably occurs through its broad anti−inflammatory effects and the regulation of miRNAs.

Quercetin: Quercetin, a bioflavonoid found naturally in many foods, is widely consumed through fruits, vegetables, and drinks such as onions, broccoli, berries, apples, grapes, tea, and red wine. It acts in multiple ways, demonstrating antioxidant, anti-inflammatory, anticancer, and anti-aging properties, as well as estrogen-like activity (48). Quercetin significantly alleviated nasal allergic rhinitis symptoms induced by ovalbumen when administered orally. Moreover, quercetin inhibited inflammation, reestablished the Th1/Th2 and Treg/Th17 balance, and disrupted nuclear factor kappa-B (NF-*κ*B) signaling in mouse models of ovalbumen. Therefore, quercetin is beneficial for reducing allergic rhinitis inflammation since it corrects these immune imbalances and inactivates NF-*κ*B (49). The results indicate that quercetin could be a promising therapeutic agent for the treatment of clinical allergic rhinitis. To better understand the impact of quercetin on Treg regulation, particularly on the targeting of FOXP3 in CRS.

Berberine: Berberine, an isoquinoline alkaloid, is a major bioactive component of the traditional Chinese medicinal herb *Rhizoma coptidis*. It possesses a wide array of pharmacological properties, including anti-inflammatory, antioxidant, lipid-lowering, glucose-lowering, and gut microbiota-modulating effects (50). Asila et al. (51) found that berberine can correct the imbalance between pro-inflammatory Th17 and anti-inflammatory Treg cells and attenuate the NF-*κ*B/JNK/RANKL signaling pathway in septic arthritis caused by *Staphylococcus aureus* in mice. Joint swelling and arthritis scores were significantly decreased in mice after berberine treatment (50, 100, or 200 mg/Kg) compared to untreated infected mice, and the percentages of Th17 (CD4+RORγt+) and Treg (CD4+CD25+FOXP3+) cells decreased significantly in synovial tissue, blood, and spleen. The same cellular changes were reflected in marked reductions in serum levels of IL-21 (associated with Th17) and TGF-*β* (associated with Treg). The molecular analysis of the lysates of both Th17 and Treg cells from the berberine-treated mice showed a significant reduction in the expression of JNK, NF-*κ*B, and RANKL – all important inflammatory mediators – suggesting that berberine directly inhibits disease progression. The role of berberine has been reported by Asila et al, but there are no reports of direct binding or modulation of FOXP3, C5aR1, or LIF. Thus, its contribution as an axis modulator is deduced from observed changes in gut and lung immune phenotype in gut-lung axis models.

### 3.2 C5aR1 and complement inhibitors

Rosmarinic acid: Rosmarinic acid is a natural ester formed from caffeic acid and 3, 4-dihydroxyphenyllactic acid. It has garnered significant interest due to its wide range of pharmacological effects, which include potent antioxidants, anti-inflammatory, antiviral, neuroprotective, and anticancer properties (52). Rosmarinic acid inhibits C5 convertase with an IC50 of 34 μM in C3b attachment inhibition, 180 μM in the classical pathway, and 160 μM in the alternative pathway (53). It is a covalent reaction between the polyhydroxylated phenyl groups of rosmamarinic acid and C3b by the formation of a thioester bond. The potential of rosmarinic acid to modulate C5aR1 remains a topic that warrants further exploration. Future research should utilize both *in vitro* and *in vivo* models to fully understand its effects and mechanisms of action.

Curcumin: Curcumin, the primary bioactive compound in turmeric, is a polyphenolic substance extracted from the rhizome of *Curcuma longa*. It possesses a diverse array of biological properties, including antioxidant and anti-inflammatory activities (54). A 241-patient randomized trial of perennial allergic rhinitis revealed that oral curcumin over 2 months had significant effects on nasal symptoms by alleviating nasal symptoms and nasal congestion through reduction of nasal airflow and inhibiting IL-4, IL-8, and tumor necrosis factor-alpha (TNF-*α*) while enhancing IL-10 (55). Further investigation is needed to determine the modulatory effect of curcumin on C5aR1 in CRS, given curcumin’s major shortcoming of low bioavailability, and to explore how to overcome this using curcumin nanoformulations.

### 3.3 LIF/JAK-STAT modulators

The LIF/IL-6 axis targets must be carefully considered, as both gp130 signaling and STAT3 activation have opposite effects on FOXP3. The therapeutic objectives consist of selective upregulation of LIF signaling (challenging, since no natural LIF receptor agonists are properly characterized), maintenance of LIF at the expense of IL-6, and context-dependent Janus kinase (JAK)-STAT regulation.

Curcumin: Curcumin suppresses JAK-STAT by several different pathways: direct binding and inhibition of JAK2, inhibition of translocation of STAT1/3 to the nucleus, and activation of SOCS1 and PIAS3 endogenous inhibitors. Curcumin repressed JAK2/STAT3 activity and increased IL-4/IL-10 in microglial cells (56). Curcumin can inversely regulate the relative effect of LIF by not only selectively suppressing IL-6-induced STAT3 and stimulating IL-4/IL-10 regulatory pathways but also increasing the relative impact of LIF.

EGCG: EGCG has a direct effect on the shared gp130 receptor: binding to gp130 (residues R382, R417, R423, S465, and N469), gp130 receptor inhibition, and downstream phosphorylation of STAT3. EGCG also decreased IL-6 in macrophages by 86% (57). This considerable suppression of IL-6 may provide a conductive environment to LIF action despite the potential concomitant activation of the LIF signal by gp130 interference. Another study revealed that EGCG modulates LIF in the inflammatory process. Akhtar and Haqqi (58) stated that EGCG changed the expression of 14% of the 80 studied proteins measured, and most significantly, it inhibited the upregulation of all 29 proteins induced by IL-1*β*, MCP-1, MIP-1*β*, and LIF. These findings open a new avenue for studying the modulatory effect of EGCG on LIF in CRS.

Apigenin: The dietary flavonoid, apigenin, may act on the activity of LIF in CRS by modulation of both the JAK/STAT and NF-*κ*B signaling pathways. LIF has been shown to activate the gp130 receptor complex by binding to gp130, leading to the phosphorylation of STAT3, which in turn, drives the transcription of pro-inflammatory genes and, consequently, pro-inflammatory activity in both apoptosis and inflammation (59). Apigenin relieves rhinosinusitis by TGF-*β*1-induced nasal mucosa remodeling by inhibiting MAPK/NF-*κ*B signaling pathways in CRS (60). Besides, apigenin regulates Th1/Th2 balance via suppression of the Th2 expression response and activation of the Th1 response to exert its anti-allergic potential in a murine model of ovalbumin-induced allergic rhinitis (61). Apigenin targets these common molecular targets and has the potential to not only inhibit the downstream effects of LIF but also to decrease LIF expression itself, providing a multi-targeted approach to inhibit LIF-driven pathology in rhinosinusitis, even without direct binding data.

## 4 Clinical evidence in sinusitis

**Figure 3:**
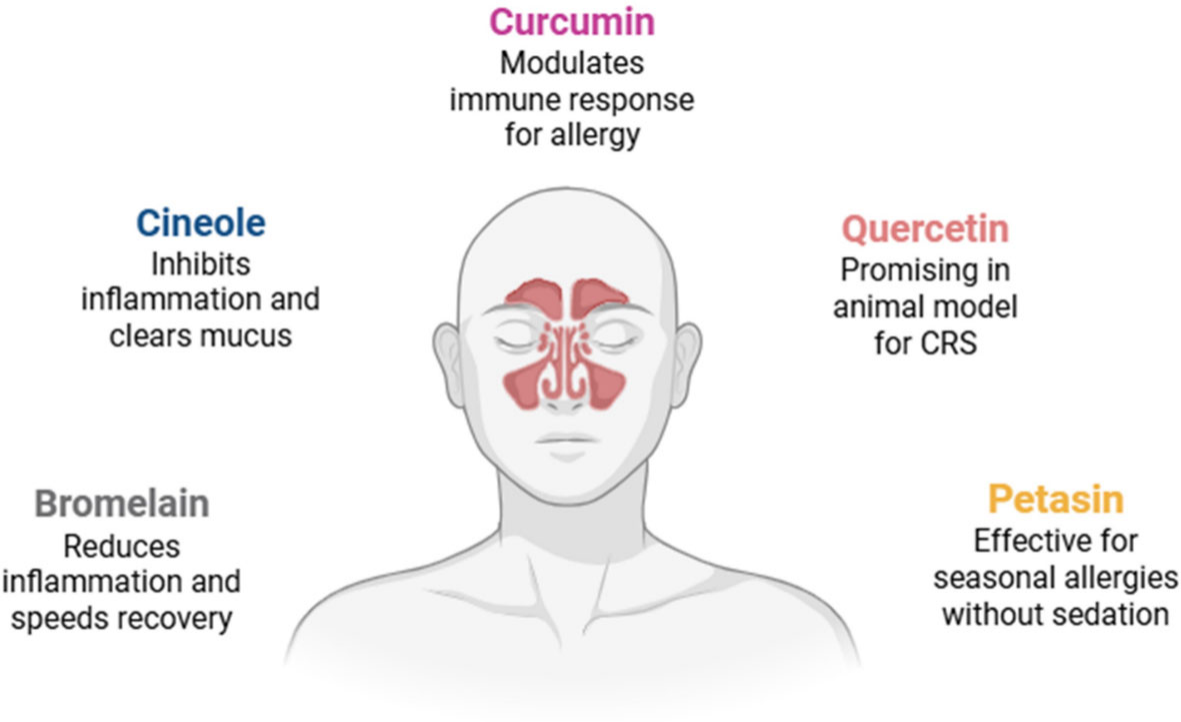
Clinical efficacy of natural compounds for clinical trials. Created with biorender.com.

The majority of clinical human data on natural compounds discussed here are from studies of acute rhinosinusitis or allergic rhinitis, not CRS. Although these diseases share some common inflammatory mechanisms with CRS, including type2 inflammation, mucosal edema, and activation of NF-*κ*B, as well as differences in chronicity, tissue remodeling, and immune dysregulation, direct transfer of the results is not possible. Thus, the studies summarized below primarily provide proof-of-concept for symptom relief, anti-inflammatory activity, and safety signals, but do not provide definitive evidence of efficacy in CRS. A growing body of clinical evidence supports the use of specific natural compounds as effective anti-inflammatory agents for sinus and respiratory conditions. These substances, including bromelain, cineole, and curcumin, target distinct inflammatory pathways and have demonstrated significant symptomatic relief across conditions ranging from acute sinusitis to allergic rhinitis, offering a complementary approach to conventional therapy (**Figure 3**). Urgent CRS trials are required to determine if these benefits extend to the chronic, often treatment-resistant sinonasal environment.

### 4.1 Curcumin

In a randomized, double-blind trial of 241 patients with perennial allergic rhinitis, 2 months of curcumin were found to improve nasal symptoms and decrease nasal airflow obstruction (*p* < 0.05) (55). Mechanistic studies showed inhibition of Th2 cytokines (IL-4 and IL-5) and pro-inflammatory cytokines (IL-8 and TNF-*α*), and increased levels of the anti-inflammatory cytokine IL-10. Inflammatory overlap provides valuable evidence when assessing allergic rhinitis rather than CRS. No specifications were provided for the curcumin formulation to improve bioavailability, and thus, the optimal dosage remained unclear. In this study, Wu and Xiao demonstrated that curcumin reduces the nasal symptoms and decreases the levels of Th2 cytokines in a chronic type 2 airway disease. But the inflammatory environment of nasal polyps in CRS may vary considerably from that of other forms of CRS.

### 4.2 Quercetin

The effect of enterotoxin B of *S. aureus* on CRS has recently been investigated in preclinical studies, including human nasal epithelial cells and several mouse models. The investigations proposed in this thesis indicate that quercetin could be a promising compound in reducing the negative impacts of CRS. It seems to do this by inhibiting remodeling of nasal mucosal tissue, decreasing inflammation, and restoring the balance between Treg and Th17 cells that are responsible for immune homeostasis. This protection may be due to the inhibition of the TLR2-NF-*κ*B signaling pathway. Based on these results, quercetin appears to be a promising therapeutic agent for the treatment of CRS, with potential benefits in reducing symptoms and enhancing patient outcomes (62). Preclinical trials are in progress, leading to human clinical trials.

### 4.3 Petasin

One hundred twenty-five patients with seasonal allergic rhinitis were selected for a double-blind, randomized study comparing the effect of butterbur extract (ZE 339, a formulation of 8 mg of total petasin per tablet, 1 tablet 4 times a day) with cetirizine (10 mg tablet once daily) over the course of 12 weeks. After two weeks, both treatments showed similar improvements on the SF-36 questionnaire and the clinical global impression scale. Two-thirds of reported adverse events for cetirizine were related to sedation (drowsiness and fatigue), whereas the adverse events for Butterbur were well tolerated (63). The researchers found that butterbur is as effective as the antihistamine cetirizine and could be a good alternative when drowsiness-causing antihistamines are not desired.

### 4.4 Adjunctive symptom−modulating natural compounds without demonstrated axis targeting

Certain natural compounds have been shown to have a clinical effect in the treatment of acute sinusitis or allergic rhinitis via a wide spectrum of mechanisms with no involvement of FOXP3, C5aR1, or LIF. These are included here only as background information to provide a full picture of the evidence and are not intended to target the disease axis or to treat CRS.

#### 4.4.1 Bromelain

Bromelain, derived from pineapple, exhibits several anti-inflammatory properties, including fibrinolysis (mucous-decomposing enzymes), regulation of bradykinin to reduce pain and swelling, suppression of cytokines such as IL-1*β*, IL-6, and TNF-*α*, and enhanced antibiotics penetration (64, 65). In a German pharmaco-epidemiological study of 116 children with acute sinusitis, monotherapy with bromelain was associated with a much shorter mean duration of recovery: 6.66 vs 7.95 days (*p* = 0.005). The scores for symptom severity improved more rapidly, especially nasal discharge and facial pain (66). Furthermore, bromelain tablets (500 FIP) used for three months (average daily dose of 3000 FIP) were evaluated in a small open-label pilot study of 12 patients with CRS and/or nasal polyps who have previously had sinus surgery, with the conclusion that bromelain appears to be well tolerated and is effective for symptom control and quality of life (SNOT-20 GAV) improvement in patients with CRS, with greater benefits in patients without nasal polyps (67).

Another study investigated the penetration of bromelain into the blood and sinonasal mucosa of patients with CRS. 20 patients received 500 mg of bromelain twice a day for 30 days, and 20 patients were in the control group. For both groups, researchers tested the turbinate and ethmoid mucosa for bromelain, as well as serum. The results indicated that bromelain was well distributed from the blood into the mucosa of the rhinosinus cavity, indicating its diffusion ability. This herb may be useful for its anti-inflammatory properties in the treatment of paranasal sinus diseases (68). Similarly, bromelain was detected in the turbinate and ethmoid mucosae after oral administration of a 500 mg bromelain tablet twice daily for 30 days in patients with CRS (Group A) and in individuals without nasal or paranasal disease (Group B). Bromelain taken by mouth has an excellent distribution from the blood to the rhinosinusal mucosa of both groups (69).

In summary, bromelain offers notable clinical benefits for both acute and chronic sinusitis, including faster recovery, improved symptom management, and enhanced quality of life, especially in patients without nasal polyps. Given its good tolerability and reliable efficacy, bromelain warrants further investigation as an adjunct treatment for sinonasal conditions.

#### 4.4.2 Cineole

1, 8-Cineole, a naturally occurring monoterpene found in plants, is frequently used to treat inflammatory diseases due to its ability to break down mucus, fight microbes, and reduce inflammation (70). A study with 152 participants of acute viral rhinosinusitis was the first to demonstrate significant improvements with cineole 200 mg, three times daily: at day 4, symptom sum scores of 6.9 ± 2.9 vs 12.2 ± 2.5 (*p* < 0.001), and at day 7, 3.0 ± 2.8 vs 9.2 ± 3.0 (*p* < 0.001), with differences on a placebo control approach with a blinded outcome (71). The single symptoms, such as headache, nasal obstruction, and secretions, improved much more quickly. Unfavorable cases were like a placebo. Cineole also enhances mucociliary clearance through mucolytic effects, as shown by reduced goblet cell numbers and downregulation of mucin genes. The high-quality RCT conducted by Kehrl et al. was conducted in patients with acute rhinosinusitis, not chronic. The effect of cineole on NF-*κ*B/Nrf2 modulation and mucolytic activity may or may not apply to CRS, and this can only be determined by future research.

Furthermore, a non-interventional survey of 350 German patients with rhinosinusitis (some also suffering from bronchitis) was conducted, in which the quality of life before and after treatment with a cineole preparation (*n* = 310) or a nasal decongestant (*n* = 40) was measured. The mean reductions in symptom frequency (64.0%), bothersomeness (52.1%), and impact (53.9%) were statistically significant over a 7-day period (*p* < 0.001), and 90.0% rated the efficacy as good/very good and quality of life as improved during work and leisure time. Based on the study results, cineole is a safe and well-tolerated treatment that was effective in improving the quality of life in rhinosinusitis (72).

To conclude, cineole is as tolerated as a placebo and produces symptom relief in acute viral rhinosinusitis and improves quality of life. While its mucolytic and anti-inflammatory properties seem logical, there is limited evidence of its efficacy in treating CRS, and it is primarily used in acute sinusitis.

## 5 Bioavailability challenges and enhancement strategies

The most promising natural polyphenols also exhibit pharmacokinetic limitations, including low solubility, rapid metabolism, and low oral bioavailability, which limit their systemic concentrations (73). To overcome these problems, improved formulation methods, including complexation and lipid-based nanocarriers, have been introduced (74, 75). One of the most valuable approaches is nasal delivery, which bypasses first-pass metabolism and utilizes mucoadhesive systems to achieve local or systemic therapeutic efficacy (**Table 2**).

**Table 2:** Natural products limitations and overcoming strategies.

Challenges	Core problem	Limitations	Overcoming strategies	Mechanism and key outcomes
Bioavailability problem	Poor pharmacokinetics of natural polyphenols hinder clinical translation.	- Poor aqueous solubility. - Rapid and extensive metabolism: Phase II glucuronidation/sulfation and first-pass hepatic metabolism. - Low oral bioavailability and short plasma half-life. - Concentration gap: Effective *in vitro* concentrations are not achievable systemically via oral dosing.	1. Absorption enhancers: - *Tool:* Adding whey protein to curcumin-piperine nanoemulsions significantly improves curcumin bioavailability (78). 2. Advanced formulation technologies: - *Tool A:* Lipid-based delivery systems (e.g., phytosomes and liposomes) (79). - *Tool B:* Nanoparticles (87).	Enhancement of natural products’ bioavailability.
Enhancement strategies	To overcome poor solubility, rapid metabolism, and low systemic exposure from oral administration.	Conventional oral doses fail to reach therapeutic blood levels.		Protect compounds, promote lymphatic absorption (bypassing first-pass), and enhance membrane permeability Nanoparticles: Increase dissolution surface area and enable transcellular transport.
Nasal delivery: bypassing systemic limitations	Systemic delivery limitations prevent achieving therapeutic concentrations at target sites.	Oral/systemic route: First-pass metabolism, systemic side effects, and failure to reach specific tissues directly.	Nasal delivery platform: Leverages the nasal cavity’s vascularized, permeable mucosa (~150–200 cm²). *Key technological formulations:* 1. Thermosensitive hydrogels 2. Mucoadhesive systems (e.g., chitosan and hyaluronic acid) (86). 3. Powder formulations (e.g., PLGA-NPs in mannitol/lactose carriers) (87). 4. Cyclodextrin-based solutions/sprays (88). 5. Device and formulation optimization.	Platform benefits: Direct target access, rapid onset, reduced systemic exposure, and non-invasive. 1. *Hydrogels:* Liquid at room temperature, gel at body temperature, extending residence time. 2. *Mucoadhesives:* Bind to mucosa, prolong contact time. Chitosan opens tight junctions and has antimicrobial properties (86). 3. *Powder/nanoparticle combinations*: Protect payload, enable deep nasal deposition and sustained local release (87). 4. *Cyclodextrin solutions:* Enable high-concentration and low-volume sprays/drops via solubility enhancement (88). 5. *Optimization:* Addressing ciliary clearance (extended residence), irritation (via buffering, osmolality control), and dose reproducibility (via standardized spray devices).

### 5.1 The bioavailability problem

Most natural compounds have serious pharmacokinetic issues that hinder bench-to-bedside translation (76). Polyphenols like curcumin, quercetin, and resveratrol have therapeutic potential but are characterized by poor solubility, extensive metabolism and bioavailability, and short half-lives, all of which pose significant challenges to clinical translation (73, 77). To achieve these pharmacokinetic benefits, it is necessary to develop innovative formulations that deliver the drug to the systemic circulation or to make structural modifications that enhance systemic delivery and drug stability.

### 5.2 Enhancement strategies

*Absorption enhancers*: Whey protein significantly improved the bioavailability of curcumin (up to 88.61%) and the antioxidant status during the intestinal phase when added to the nanoemulsions containing a curcumin-piperine complex. While the addition of whey protein, especially at 5% concentration, led to an enhancement in the functional performance and bioavailability of the nanoemulsions, the curcumin-piperine complex without whey protein gave the smallest particle size and best physicochemical stability (78).

*Technologies of formulation*: Poorly absorbed phytochemicals are protected from degradation, enhanced by intestinal lymphatic transport, reduced by first−pass metabolism, and extended by prolonging circulation time by lipid−based formulations like phytosomes, liposomes, and solid lipid nanoparticles (79, 80). In the case of curcumin, a phytosome complex (Meriva^®^) enhanced the total curcuminoid absorption by ~29-fold compared to the unformulated equivalent of curcumin (79). Likewise, liposomal formulations of quercetin have been shown to improve bioavailability: a recent systematic review found that liposomal quercetin increases absorption (81, 82). These formulations can be made into nanoparticles, which will help reduce particle size, increase dissolution surface area, and enhance transcellular transport, thereby improving absorption and systemic exposure.

### 5.3 Nasal delivery: bypassing systemic limitations

The benefits of direct nasal delivery are many, and this route could be an attractive option for medication delivery. Most importantly, it provides direct access to the area to be treated and allows higher drug concentrations in the affected area, which may be hard to achieve using systemic approaches. Furthermore, this method can avoid the first-pass metabolism problem, as the drug does not get significantly metabolized before it reaches the site of action (83). Nasal mucosa has a rich blood supply, facilitating rapid drug absorption and a rapid onset of action. This also reduces systemic exposure, thereby significantly reducing off-target issues associated with conventional systems. Moreover, the nasal route is more convenient for patients, avoiding invasive procedures and thus being more patient-friendly. Importantly, the sino-nasal epithelium has a significant absorption surface area of approximately 150–200 cm^2^, which helps to maximize the efficacy of nasal drug delivery (84, 85).

*Technologies in nasal formulations*: A thermosensitive hydrogel is a liquid that is easy to administer at room temperature and forms a gel at 37 °C, increasing residence time. Mucoadhesive systems based on chitosan, hyaluronic acid, or carbomer bind to the nasal mucosa by way of electrostatic forces, increasing the contact time and increasing absorption. Chitosan has other advantages: biodegradation, biocompatibility, temporary opening of tight junctions that enhance paracellular transport, and natural antimicrobial effects (86). Poly (lactic acid-co-glycolic acid) (PLGA) nanoparticles are incorporated into mannitol/lactose microparticles, enabling nasal powder delivery of the compounds they contain and achieving high local concentrations (87). Solutions made with cyclodextrin can be easily delivered as nasal drops/sprays, and the high solubility allows therapeutic concentrations to be produced in low volumes (88). Some compounds may cause nasal irritation; they must be formulated appropriately using buffering, osmolality control, and concentration optimization. Patient education and standardized spray devices with dose counters enhance the reproducibility of delivery (89). To conclude, the effective nasal delivery of drug products requires the use of *in situ* gel-forming thermosensitive polymers in combination with mucoadhesive polymers (such as chitosan, hyaluronic acid, or carbomer) to increase the drug’s contact time with the mucosa and drug absorption, with chitosan also providing temporary tight-junction opening, biodegradability, and antimicrobial effect. High local drug concentration is possible with advanced carriers, such as PLGA nanoparticles in mannitol/lactose microparticles for dry powder formulations and cyclodextrin-based formulation solutions for low-volume sprays. To achieve tolerability, it’s essential to use the right buffer, maintain osmolality control, optimize concentration, provide patient education, and use dose-counting devices.

## 6 Comparison of natural compounds and conventional therapies for treatment of sinusitis

The CRSwNP treatment options currently used (such as corticosteroids and biologics) are only effective for symptom management but do not modify the underlying disease, leading to symptom recurrence once therapy is stopped (90). Instead, the targeted natural compounds represent a paradigm shift, targeting the underlying defects in immunity, including Treg deficiency and complement errors, and have the potential to achieve long-term disease modification at reduced cost, although with less comprehensive clinical evidence (**Table 3**).

**Table 3:** Comparative treatment analysis of natural products and conventional sinusitis therapies.

Features	Corticosteroids (94, 102, 103)	Biologics (95, 96, 98, 104)	Natural compounds (43, 47, 51, 66, 71)
Mechanism	Non-specific inflammatory suppression	Targeted Type 2 blockade	Multi-target immune regulation
Target level	Downstream inflammation	Downstream Type 2 effectors	Upstream regulatory mechanisms
Efficacy evidence	Multiple large RCTs	Multiple large RCTs	Preclinical strong Clinical preliminary
Disease modification	No—symptoms recur	No—symptoms recur	Potential—may restore tolerance
Safety	Local irritation; systemic concerns	Injection reactions; conjunctivitis	Generally favorable
Endotype specificity	Non-specific	Type 2 only	Potentially broader

### 6.1 Current standard of care

Intranasal corticosteroids are the initial pharmacotherapy and inhibit inflammation by transcriptionally repressing the glucocorticoid receptor (91). The anti-inflammatory effects of intranasal corticosteroids include decreased vascular permeability as well as inhibition of the release and/or formation of mucous secretogogues (eg, histamine, leukotrienes, prostanoids, and platelet-activating factor) (92). The effects are believed to be due to inhibition of the release of pro-inflammatory mediators, including adhesion molecules, cytokines, mast cells, basophils, and eosinophils. The intranasal corticosteroid binds to the glucocorticoid receptor in the cytoplasm, forming a complex that leads to a decrease in the amount of proinflammatory molecules and cells (93). Intranasal corticosteroids have a well-established safety profile with predominantly local (e.g., epistaxis, headache) events, which are often mild to moderate (94). Newer intranasal corticosteroids should continue to be used in children, and continued monitoring is recommended.

In the last two decades, the landscape of severe asthma, atopic dermatitis, and nasal polyp CRS has been revolutionized by the development of targeted therapeutics that target specific inflammatory pathways, particularly those associated with the T2 inflammatory pathway, which have largely failed to be effective in conventional treatment. Dupilumab is a fully human monoclonal antibody targeting both IL-4 and IL-13 signaling and has been shown to be effective in several type-2 driven diseases, but is often associated with ocular surface disorders that include conjunctivitis, blepharitis, keratitis, dry eye, and eye pruritus, which typically respond to topical treatment (95, 96). Rare but serious allergic/eosinophilic complications, like paradoxical eosinophilia, have been reported with allergic conjunctivitis (97). An anti−IgE antibody, omalizumab, carries a boxed warning for anaphylaxis and must be administered in a healthcare facility prepared for resuscitation, but long−term studies do not show a definite association with cardiovascular events or malignancy (96). Mepolizumab, an anti−IL−5 agent, is paradoxically linked to an asthmatic crisis reporting odds ratio of 108.04 (98). These are all very well-tolerated agents when used together, but have some unique side-effect profiles — for example, omalizumab can cause anaphylaxis, mepolizumab can cause respiratory decompensation, and dupilumab can cause ocular events — so careful patient selection and careful monitoring are important to optimize the benefit/risk ratio of the use of biologic agents.

### 6.2 Natural compounds: potential advantages and limitations

*Strengths:* Natural products like EGCG, curcumin, quercetin, resveratrol, rosmarinic acid, and berberine influence biological pathways by inhibiting DNA methyltransferases, the complement cascade, and JAK-STAT signaling. Furthermore, clinical evidence supports the effectiveness of bromelain and cineole in treating acute sinusitis, while additional compounds are demonstrating emerging evidence of benefit (as discussed in Sections 3 and 4).

*Limitations:* Evidence quality gaps in biologics are based on many well-designed RCTs with objective endpoints and long-term follow-up, whereas evidence for natural compounds is based on numerous preclinical studies, small pilot trials, and studies on related diseases. Oral administration of natural compounds and achieving therapeutic tissue levels remain challenging despite efforts to improve it (99, 100). Variability in the products used to produce botanical supplements makes it difficult to use them clinically. The immune-modulating natural compounds likely take weeks to months to rejuvenate Tregs and rebalance inflammation, a process that is longer than the immediate healing effect of corticosteroids (101). The disparity in CRS endotypes suggests that not every patient is a responder; Type 2-high patients with reported Treg deficiency and complement activation may be ideal candidates, while other subgroups may show slight improvement.

## 7 Future directions and research priorities

### 7.1 Clinical trial design

*Priority 1*: CRS patients stratified by phenotype (CRSwNP vs CRSsNP; Type 2 vs non-Type 2 endotypes) will receive priority in randomized, double-blind, placebo-controlled trials. FOXP3+ cell tissue baseline measurement, complement activation (C5b-9 and C5aR1), and IL-6/LIF ratios. Validated results, such as SNOT-22 (a validated questionnaire used by rhinologists to measure symptoms and quality of life in patients with sinus disease), endoscopic scores, CT score, and objective olfaction test. At least a 3–6 months period to determine the long-term benefit, 12 months of follow-up after stopping to determine disease modification.

*Priority 2*: Dose-finding and bioavailability research, including pharmacokinetic samples involving the plasma and tissue levels. Compare bioenhancer formulations with standard ones. Determine low dosages of efficacy. Evaluate intranasal and oral administration efficacies. Furthermore, investigate the combinatorial benefits of natural compounds with corticosteroids or biologics.

*Priority 3*: Mechanism of action verification using serial tissue biopsies (pre-, during-, and post-treatment) of FOXP3+ Tregs, complement-specific markers, and cytokines: This aims to verify the disease mechanism of action. Functional assays and peripheral blood Tregs phenotyping. Clinical response correlated with mechanistic changes, demonstrating that natural compounds bind to the molecular targets.

### 7.2 Combination strategies and advanced delivery

*Rational combinations*: Full-network multi-targeting of FOXP3 (EGCG and resveratrol), C5aR1 (rosmarinic acid and curcumin), and LIF/JAK-STAT (curcumin and apigenin). Factorial designs that compare combinations of elements would determine synergies. Add-on trials of natural compounds as adjuncts to intranasal corticosteroids or biologics may demonstrate superior efficacy and help reduce the dose of more costly compounds.

*Sophisticated nasal dosage formulations*: Thermosensitive *in situ* gels, nanoparticle-in-microparticle powders, and mucoadhesive patches could allow once or twice a day dosage misadministration. By conjugating antibodies to nanoparticles that can target specific cell types (CD4 to T cells; CD88 to C5aR1-expressing cells), compounds can be concentrated in inflamed tissue and a better therapeutic index achieved.

### 7.3 Biomarker-guided selection and mechanistic research

*Predictive biomarkers*: Patients who have reported low tissue or circulating FOXP3+ Tregs are ideal targets for FOXP3-enhancing interventions. Tissue C5b-9 deposition, serum C5a, or C5aR1 expression on T cells may be used to identify patients who are most at risk of responding to complement-inhibiting compounds. If the assays reveal that IL-6 levels are higher than LIF levels, this specific group may be highly attractive for IL-6-inhibitory substances. Transcriptomic endotype-based classifiers of blood would also allow matching patients to therapy with the highest likelihood of being effective for the specific pattern of inflammatory activity.

*Critical research gap*: There are very few studies directly investigating LIF, LIF receptor, and LIF signaling in CRS tissue. Among the research priorities, quantification of tissue LIF levels in CRSwNP, CRSsNP, and control groups, estimation of IL-6/LIF ratios and their associations with Treg deficiency, and the feasibility of exogenous LIF to replenish Treg populations in CRS tissue explants must be considered. Future studies should quantify LIF, IL-6, and FOXP3 levels in the tissues of CRSwNP versus CRSsNP and determine the relationship between cytokine expression and Treg numbers. In addition, exogenous LIF could be evaluated in CRS tissue explants to determine whether it restores Treg populations.

## 8 Discussion

### 8.1 Paradigm shift: from symptom suppression to immune tolerance restoration

The existing CRS management aims to inhibit inflammatory mediators without targeting the underlying immune dysregulation (105), thereby leading to relapse of the disease upon therapy withdrawal. The FOXP3-C5aR1-LIF axis is a mechanistically discrete therapeutic target of immune regulation at its epicenter (30, 106, 107). FOXP3+ Tregs are grandiosers; in other words, their absence allows effector responses to be uncontrolled (108). Complement signaling C5aR1 inhibits Treg induction *in vivo* and generates micro-expands that maintain regulatory cell destruction (109). When the balance between LIF and IL-6 is disturbed, the former stimulates FOXP3 expression and regulates the phenotype (26). The activation of this regulatory axis has potential for disease-modification implementation — restoration of immune tolerance that leads to long-term remission following therapy withdrawal.

Natural compounds are helpful tools for testing this change of paradigm, employing core cellular pathways, such as epigenetic regulation, receptor antagonism, and modulation of signal transduction, to reestablish regulatory capabilities rather than merely suppressing inflammation (43, 47, 51, 66, 71). The mechanistic interconnection is an ardent supporter of multi-target intervention. Treg deficiency occurs due to complement activation via C5aR1, which suppresses the induction of FOXP3; LIF suppresses the Th17-stimulating effects of IL-6, whereas it stimulates Tregs (26). The problem with single-target interventions is that they may not be sufficient; for example, in complement-mediated blockade, the LIF/IL-6 balance, or the direct promotion of FOXP3, simultaneous intervention across the network is required.

### 8.2 Evidence gaps and translation challenges

The major weakness is the lack of high-quality clinical evidence with specific reference to CRS. Although the rationale is quite solid and the preclinical evidence is impressive, well-designed RCTs in target populations are needed at the base of the evidence pyramid. Existing research is based on allergic rhinitis (similar but not identical), acute sinusitis (with different temporal effects), small pilot trials, and preclinical models, which may not be relevant to the complexity of human CRS.

Conducting stratified efficacy trials in a CRS population requires validated outcomes, appropriate sample sizes, and adequate duration. These trials are challenging because natural products are usually unpatentable, which lowers commercial interest, and because the diverse population requires larger sample sizes. However, there is a possibility of making payoff-disease-modifying therapy available to large populations at affordable rates, which is worth investing in. Pragmatic measures have been taken, such as building on available safety data, adding-on trials (which are ethically less challenging than monotherapy comparisons), adaptive designs that identify responsive subpopulations most efficiently, and nasal formulations, which offer clear intellectual property for commercial development.

### 8.3 Safety and economic considerations

Natural preparations also tend to have a better safety profile than long-term corticosteroids or potent immunosuppressants. Many of them possess long histories of dietary consumption or traditional medicine. The safety is not absolute, though: drug-herb interactions (piperine is CYP450 inhibitor and bromelain increases the effect of anticoagulants), the problems of quality/contamination (heavy metals, pesticides, microbial toxins, and undeclared pharmaceuticals), the concerns of the specific chemicals (raw butterbur hepatotoxicity), and the risks in relation to the population (pregnancy/nursing, children, and immunocompromized) are to be considered (110–114).

*The risk-benefit analysis seems to be good:* Safer than long-term systemic corticosteroids (no hypothalamic–pituitary–adrenal suppression, no osteoporosis, and no hyperglycemia); safer than surgery (there is no risk of anesthesia, no bleeding, and no structural damage); possibly safer than biologics (no profound immunosuppression), much safer than expensive (expanding access). It is not correct to compare it to no treatment; rather, it should be compared to the existing standard of care, where the natural compounds might have better risk-benefit profiles.

*Economic factors are overwhelming:* CRS in the USA is estimated to cost the USA $10–13 billion per year in direct costs. Also, the indirect costs related to the losses of work productivity due to CRS are estimated at more than $20 billion each year (115). The Food and Drug Administration has approved several new therapies for the treatment of CRSwNP; however, there is potential for financial burden on patients and payers due to the high costs of these new therapies. The cost of CRSwNP treatments ranged from $7050 for EDS‐FLU to $28, 324 for dupilumab for commercially insured patients. The cost of these conditions was between $1036 for other drugs and $3549 for mepolizumab (116). Natural compounds can save money by reducing the extent of absences from work due to disease-related conditions. They also help reduce medical costs by reducing exacerbations, emergency visits, and surgeries. Moreover, they help achieve global health equity by lowering drug prices, particularly in low- and middle-income countries where biologics tend to be costly.

## 9 Conclusion

CRS is a significant burden on public health, driven by immune dysregulation rather than mere infection or allergy. Clues point to a lack of FOXP3+ regulatory T cells, the complement system being activated by C5aR1, actively preventing Treg induction, and a disequilibrium between the LIF and IL-6 axes, with pro-inflammatory and regulatory signaling. These three molecular targets are regulatory network nodes that are interconnected and govern immune tolerance and inflammation. Existing interventions address end effects but do not address the underlying dysregulation, leading to disease recurrence and the need to treat it throughout the rest of their lives.

EGCG, curcumin, quercetin, resveratrol, rosmarinic acid, berberine, bromelain, and cineole are a number of natural anti-inflammatory compounds that modulate the FOXP3–C5aR1–LIF axis in various ways: epigenetic regulation through inhibition of DNMTs, interference with the complement cascade by changing the C3b and its receptor, control of JAK-STAT signaling through shifting the LIF/IL-6 balance, and direct actions. The preclinical data supporting the validity of these mechanisms are strong; *in vitro* data show that the targets can be engaged at physiologically relevant concentrations, while animal models show efficacy *in vivo*.

The CRS clinical evidence is particularly sparse yet encouraging. Bromelain is highly useful in acute sinusitis and has preliminary usefulness in chronic illness. Cineole has strong efficacy in acute rhinosinusitis, with well-designed RCTs supporting its use. Curcumin improves outcomes in allergic rhinitis, with immunological alterations in line with Treg enhancement. Quercetin lowers inflammation in preclinical CRS models and enhances sinonasal epithelial performance. There are promising results when combination natural product formulations are used in small trials.

The future needs include RCT in phenotyped CRS groups, bioavailability maximization (via the use of advanced formulations, especially intranasal delivery systems), exploration of synergistic combinations that can address multiple nodes of a network at once, the paradigm of biomarker-directed patient selection and response monitoring, and translation of emerging evidence into clinical practice. Nevertheless, the potential impact outweighs the cost, despite challenges such as a lack of commercial motivation, the heterogeneity of diseases that require large sample sizes, and the need for quality assurance education. Provided that natural compounds to the FOXP3-C5aR1-LIF axis can be demonstrated to possess the capability of restoring immune tolerance besides disease modification and not symptom suppression, it would be a paradigm shift in the management of CRS, with the added benefits of safety, cost-effectiveness, and multi-mechanism action over preexisting treatments, and also enabling more patients across the globe to gain access to effective therapy.

The scientific basis exists, the mechanistic explanation is logical, and the initial data are promising. The next step is to conduct rigorous translational studies to determine whether these potential benefits translate into real clinical improvements for patients with CRS. A combination of mechanistic immunology, phytochemical pharmacology, and intensive clinical research offers the opportunity to go beyond symptom management to achieve actual immune control and disease management in this widespread and debilitating pathology.
